# ﻿Three new species of non-marine ostracods (Crustacea, Ostracoda) from small water bodies of northern China

**DOI:** 10.3897/zookeys.1097.79713

**Published:** 2022-04-29

**Authors:** Na Yu, Shunxin Ma, Qianwei Wang, Dayou Zhai

**Affiliations:** 1 College of Teacher Education, East China Normal University, Shanghai, China; 2 Fengxian High School, Shanghai, China; 3 Yunnan Key Laboratory for Palaeobiology, Institute of Palaeontology, Yunnan University, Kunming 650500, China; 4 MEC International Joint Laboratory for Palaeobiology and Palaeoenvironment, Yunnan University, Kunming 650500, China

**Keywords:** biodiversity, *Cyclocypris*, freshwater ecosystem, *Pseudocandona*, taxonomy, *Tonnacypris*

## Abstract

Three new species, *Pseudocandonacheni***sp. nov.**, *Cyclocyprispangi***sp. nov.**, and *Tonnacyprisrectangularis***sp. nov.**, collected from northern China, are described in this study. *Pseudocandonacheni*, of the *compressa* group, is difficult to be distinguished from other members of the same group by carapace morphology alone, but can be readily recognised by the structure of the hemipenis comprised of a long lobe *a*, distally inflated lobe *h*, and exteriorly pointed lobe *b*, as well as thick trunks of the male fifth limb endopodites. *Cyclocyprispangi*, despite its similarity in carapace morphology to its congeners, can be identified based on the morphology of the hemipenis, which bears a slender, slightly curved lobe *h*, and an S-shaped process accompanying lobe *b*, in addition to the rectangular trunk of the male fifth limb endopodite. On the other hand, *Tonnacyprisrectangularis*, described on the basis of females only, can be distinguished from other *Tonnacypris* Diebel & Pietrzeniuk, 1975 representatives by its distinct sub-rectangular carapace alone. Other differences include the length of the swimming setae, the length of the distal claws on the second antennae, and the morphology of the pincer organ on the seventh limb. This study contributes to the poorly known extant non-marine ostracod fauna of Inner Mongolia and Beijing, and generally to the central-eastern Palaearctic region. In addition, the known distribution range of *Tonnacypris* is extended eastwardly by *T.rectangularis*. The valve-morphology data are useful for identifying fossil/sub-fossil representatives.

## ﻿Introduction

Taxonomic studies of the extant non-marine ostracods of China lag behind other Eurasian regions where ostracods have been extensively investigated, such as Europe (e.g., Meisch 2000), Japan (e.g., Okubo 2004; Smith et al. 2011), Korea (e.g., Karanovic and Lee 2012), and Thailand (e.g., Savatenalinton and Suttajit 2016). More than 90 years after the pioneering work of Sars (1903) and the first taxonomic study by a native ostracod worker (Chen 1957), only 47 species of extant non-marine ostracods were known from China based on the checklist by Yin and Martens (1997). Yu et al. (2009) compiled an updated checklist of 154 extant, non-marine ostracod species from China, but only 94 of these were based on the living material (Martens and Savatenalinton 2011). Yu (2014) has provided the most comprehensive taxonomic description of the extant ostracods of China to date, which included 91 species, the majority of which have soft-part morphology described. The number of species presented by Yu (2014) is in accordance with the Martens and Savatenalinton (2011) estimate. Recently, a number of publications added taxa to the catalogue of the living non-marine ostracods of China (e.g., Zhai and Xiao 2013; Kong et al. 2014; Zhai and Zhao 2014; Ma and Yu 2018, 2020; Peng et al. 2021), and the total number may be slightly more than 100. Probably many more species await discovery and description, considering the comparatively small number of taxonomic works done in China since Sars (1903) and Chen (1957), and disparity of geographical settings with diverse aquatic ecosystems found across the vast land area of this country. A number of ostracods recorded during the studies of the valve material from the superficial sediments have been left in open nomenclature (e.g., Mischke et al. 2003, 2007; Zhai et al. 2013; Li et al. 2021). Even in some taxonomic publications, species were left with open nomenclature due to the incomplete illustrations and descriptions. For example, in the discussion on the identification methods of ostracods of Zhai et al. (2017), a number of unidentified species were mentioned, with only some limb-structure measurements provided. In addition, several species, although named, are known only after empty valves without information on the soft parts (Yang et al. 1982; Yang and Huang 1983; Huang et al. 1985), hindering further understanding of their ecology and phylogeny. Therefore, there is an urgent need to accumulate more taxonomic data on the living non-marine ostracods of China, preferentially based on both carapace and soft-part morphology, to facilitate the application of ostracods in various fields of scientific research including palaeoclimatology, ecology and biology.

In this study, we present detailed taxonomic descriptions of three unnamed species originally reported by Zhai et al. (2017), where only some limb structures had been measured and no illustrations of their carapace or soft parts were presented. We also provide a brief description of the ecological characterisation of these species. This work will provide valuable information for future research based on ostracods, especially in Beijing and Inner Mongolian areas.

## ﻿Material and methods

Ostracods were collected from four sites (Table 1). At the sites Y11, Y26, and Y30, bottom substrates were collected with a simplified sucking device modified from Viehberg (2002), and were sieved with a mesh of 0.15 mm. The sample Y34 was collected by sieving the detritus-rich substrate and the macrophytes with a mesh of 0.15 mm. The samples were fixed with 70% ethanol after excess water was drained. Electrical conductivity of the ambient water was measured with a HANA HI98128 device.

**Table 1.** Information on sampling sites (GPS coordinates based on WGS84 system).

Site	Coordinates	Habitat description	Date	EC	Specimens
Y11	43°22'26.0"N, 116°44'36.8"E	pond with abundant plant detritus, formed in rechanneled bed of Gongger River, Hexigten Banner, Inner Mongolia	12.v.2015	2314	dyzoc575‒580, dyzoc819, dyzoc821 (*Tnr*)
Y26	43°00'20.5"N, 115°47'34.9"E	small pond with abundant plant detritus, connected with small creek in Zhenglan Banner, Inner Mongolia	18.v.2015	458	dyzoc567‒570, dyzoc706‒707, dyzoc813 (*Psc*)
Y30	42°58'55.8"N, 115°49'14.0"E	small swamp with abundant grass in Zhenglan Banner, Inner Mongolia	18.v.2015	562	dyzoc625, dyzoc626, dyzoc814 (*Psc*);
					dyzoc675, dyzoc816‒817 (*Clp*)
Y34	between 40°33'59.7"‒34'11.5"N, 116°47'9.7"‒48'25.8"E	pond with a few macrophytes, flowed through by mountain brook in vicinity of Beijing	25.v.2015	199	dyzoc558‒563 (*Clp*)

**Key**: EC, electrical conductivity in µS cm^‒1^. *Clp*, *Cyclocyprispangi* sp. nov.; *Psc*, *Pseudocandonacheni* sp. nov.; *Tnr*, *Tonnacyprisrectangularis* sp. nov.

In the laboratory, samples were transferred to a Petri dish, from which ostracods were picked under the Olympus SZX16 stereomicroscope and then stored in 70% ethanol in centrifuge tubes. Soft parts of the specimens were dissected with a pair of sharpened, fine needles attached to bamboo handles, sealed in Hydro-Matrix (Micro-Tech-Lab, Graz, Austria) and drawn with the aid of a camera lucida attached to the Olympus CX31RTSF microscope. Carapaces were stored dry on the micropalaeontological slides. Carapaces and valves that are illustrated were coated with gold and imaged under the JEOL 5800 LV, or the FEI Quanta 200 scanning electron microscope (SEM). All specimens are deposited at the Yunnan Key Laboratory for Palaeobiology, Institute of Palaeontology, Yunnan University.

### ﻿Terminology and abbreviations

**A1** antennule;

**A2** antenna;

**Hp** hemipenis;

**L5** fifth limb;

**L6** sixth limb;

**L7** seventh limb;

**LV** left valve;

**Md** mandible;

**Mx** maxillula;

**RV** right valve;

**UR** uropodal ramus.

Terminology of the limb chaetotaxy follows Broodbakker and Danielopol (1982), Martens (1987) and Meisch (1996, 2000). Terminology of the structures of reproductive organs follows Danielopol (1969, 1978). Systematics follows Meisch et al. (2019).

## ﻿Taxonomy

### ﻿Suborder Cypridoidea Baird, 1845

**Family Candonidae Kaufmann, 1900**

**Subfamily Candoninae Kaufmann, 1900**

#### Genus *Pseudocandona* Kaufmann, 1900

##### ﻿ Pseudocandona cheni sp. nov.

Taxon classificationAnimaliaPodocopidaCandonidae

938C6777-21D1-504B-A873-F781777834EE

http://zoobank.org/1023A7A8-5811-4B6E-8E8A-062596F1BA7B

Figs 1 , 2 , 3

Pseudocandona sp. 2 – Zhai et al. 2017: 486, fig. 9.

###### Type locality.

A small shallow pond (Y26, Table 1) in Inner Mongolia, China.

###### Type material.

***Holotype***: one male (dyzoc567). ***Allotype***: one female (dyzoc569). ***Paratypes***: one male (dyzoc568) and three females (dyzoc570, dyzoc706, dyzoc707). All from the type locality, with soft parts dissected, valves preserved on the micropalaeontological slides.

###### Other material.

One male (dyzoc625) and one female (dyzoc626), both from the site Y30 (Table 1), with soft parts dissected, valves preserved on the micropalaeontological slides. One female (dyzoc813), from the type locality. One female (dyzoc814), from the site Y30 (Table 1). Both undissected, with carapace enclosed, preserved on the micropalaeontological slides.

###### Etymology.

This species is named after Prof. Shouzhong Chen (= Shoutsung Chen; Institute of Hydrobiology, Chinese Academy of Sciences), who was the first among the Chinese ostracod workers to describe soft parts of non-marine ostracods from China (Chen 1957).

###### Dimensions.

Male, *n* = 3, LV, length 860‒872 μm, height 510‒535 μm; RV, length 842‒849 μm, height 485‒513 μm. Female, LV, *n* = 5, length 874‒941 μm, height 526‒574 μm; RV, *n* = 3, length 874‒903 μm, height 497‒520 μm.

###### Diagnosis.

**Figure 1.**
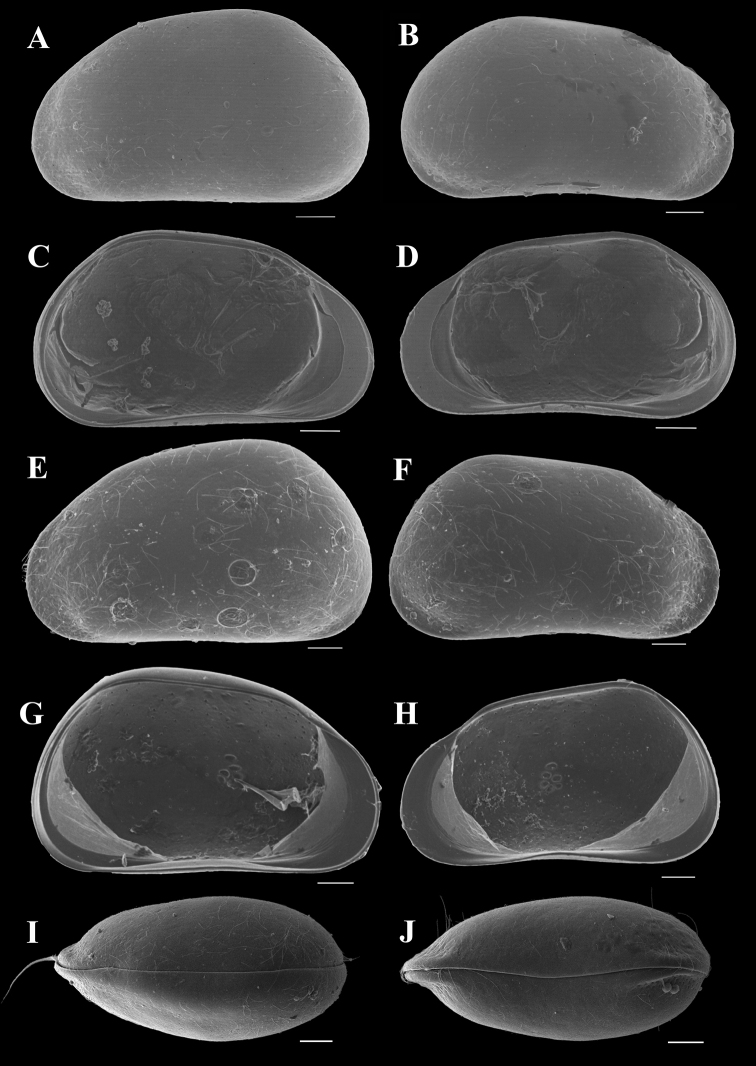
*Pseudocandonacheni* sp. nov. **A–D** male, dyzoc567 (holotype) **A** outer view of LV**B** outer view of RV**C** inner view of LV**D** inner view of RV**E–H** female, dyzoc569 (allotype) **E** outer view of LV**F** outer view of RV**G** inner view of LV**H** inner view of RV**I** female, dyzoc813, dorsal view of carapace, anterior to left **J** female, dyzoc814, ventral view of carapace, anterior to left. Scale bars: 100 μm.

**Figure 2.**
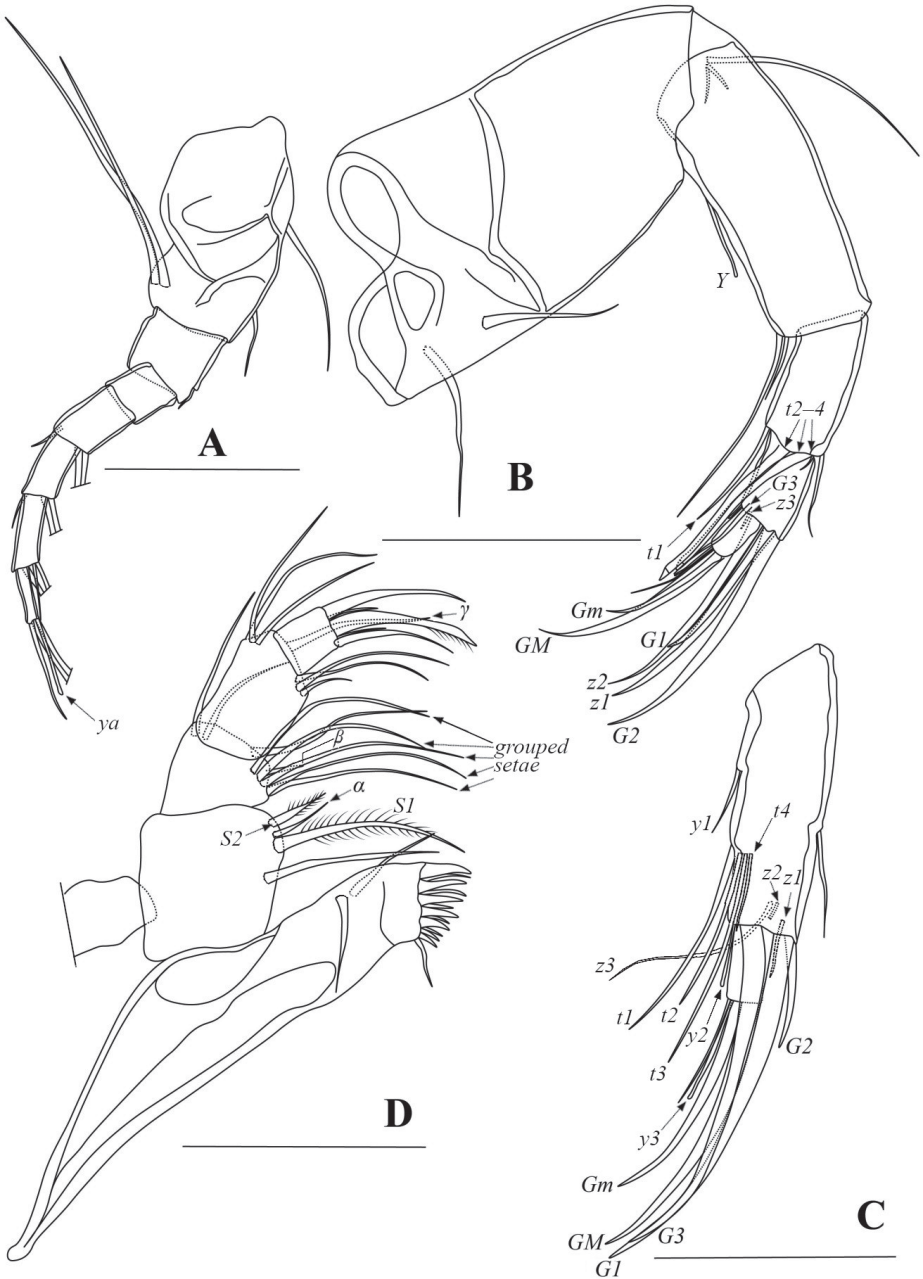
*Pseudocandonacheni* sp. nov. **A** male, dyzoc567 (holotype), A1**B** male, dyzoc567, A2**C** female, dyzoc570, part of A2**D** male, dyzoc567, Md. Scale bars: 100 μm.

**Figure 3.**
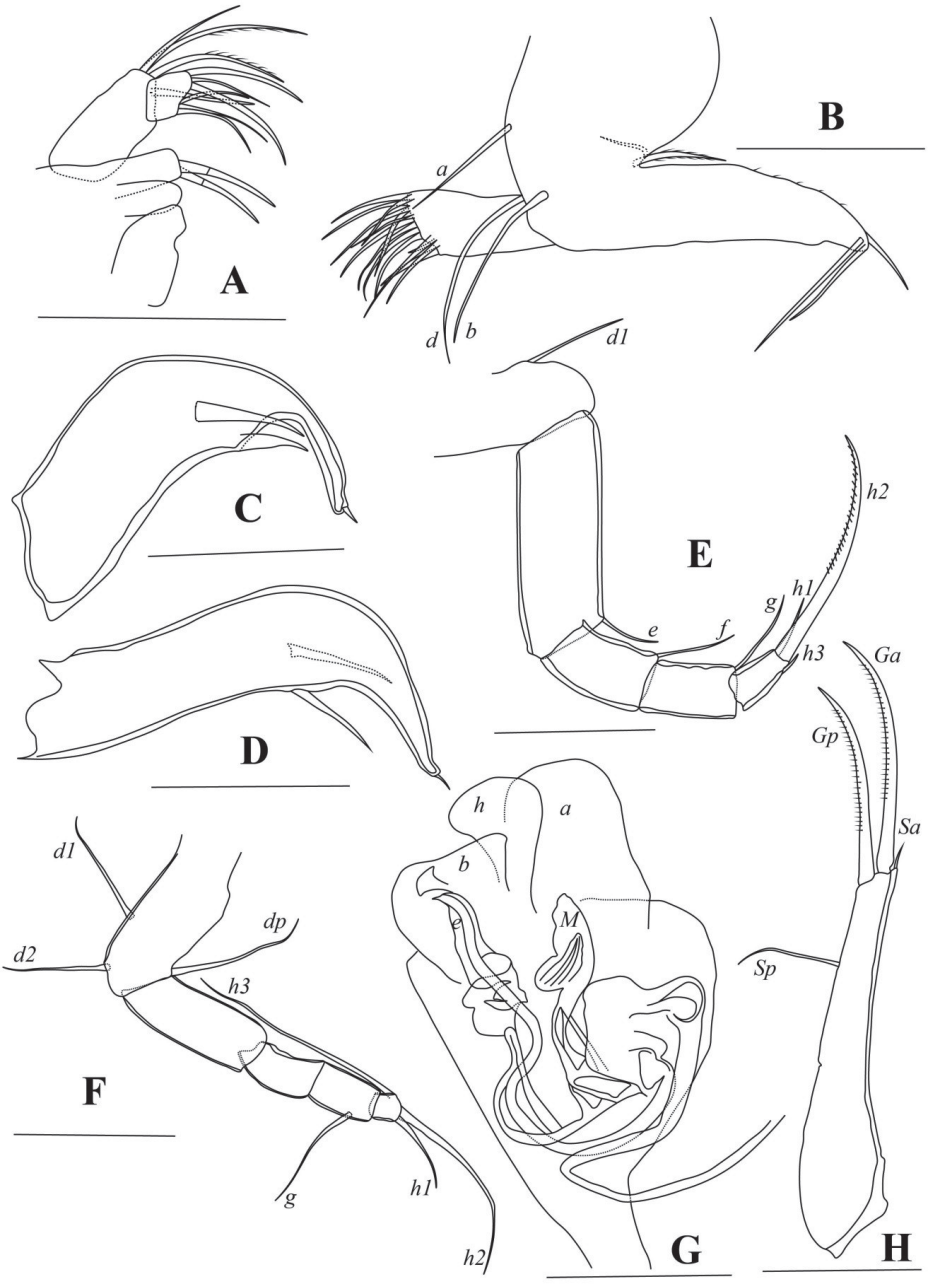
*Pseudocandonacheni* sp. nov. **A** male, dyzoc567 (holotype), Mx**B** female, dyzoc570, L5**C** male, dyzoc567, right L5 palp **D** male, dyzoc567, left L5 palp **E** male, dyzoc567, L6**F** male, dyzoc567, L7**G** male, dyzoc567, Hp**H** male, dyz567, UR. Scale bars: 100 μm.

Carapace sub-trapezoidal in lateral view, relatively short and stout, with hinged part of dorsal margin nearly straight and sloping anteriorly (Fig. 1). Setal group on second segment of Md palp with five setae (Fig. 2D). Right palp of L5 with wide trunk and ventrally curved finger-like end (Fig. 3C). Left palp with elongated and ventrally curved trunk (Fig. 3D). Hemipenis with M-process triangular distally. Lobe *a* tongue-like. Lobe *b* shortest, with sub-quadrate distal part on inner edge and triangular distal part on outer edge. Lobe *h* with rounded distal part (Fig. 3G).

###### Description.

Carapace surface densely covered with small shallow pits in anterior, posterior, and dorsal areas (Fig. 1B). In lateral view, greatest height posterior of mid-length. Postero-dorsal angle of male valve (Fig. 1A, B) slightly blunter than female (Fig. 1E, F). Ventral margin nearly straight. Anterior margin narrower than posterior. Anterior calcified inner lamella wide. Carapace compressed in anterior area and postero-ventral corner (Fig. 1I, J). LV overlaps RV on dorsal, ventral, and posterior sides, and slightly exceeds RV anteriorly (Fig. 1I, J).

A1 (Fig. 2A) seven-segmented. First segment with two dorsal and two long ventral setae. Second segment with one short dorso-apical seta. Third segment without seta. Fourth and fifth segments with two long dorso-apical setae and one short ventro-apical seta, respectively. Sixth segment with one short and three long apical setae. Terminal segment with one short and two long setae and aesthetasc *ya*.

Male A2 (Fig. 2B) five-segmented. Seta *t2* and *t3* transformed into male bristles, both similar in morphology, each terminating with slightly inflated, triangular process. Claws *z1* and *z2* long, slightly shorter than claw *G2*. Seta *G3* slim, slightly exceeding end of terminal segment. Claw *G1* short, slightly exceeding half-length of claw *G2*. Claw *Gm* slightly exceeding half-length of claw *GM*.

Female A2 (Fig. 2C) four-segmented. Claw *G2* short, not reaching half-length of claw *G3*. Claw *G3* slightly shorter than claw *G1*. Claw *Gm* exceeding half-length of claw *GM*. Setae *t1*‒*4* unequally long, with *t1* and *t3* being longest while *t4* being shortest. Seta *z1* short, extending to ca. mid-way of terminal segment. Setae *z2* and *z3* extending to ca. mid-way of *G*-claws but *z2* slightly longer than *z3*.

Md (Fig. 2D) palp with short and slender *α*-seta. Seta *β* short and slender with 5 grouped setae and one sub-equally long accompanying seta on second segment. Seta *γ* long, smooth, and slender.

Mx (Fig. 3A) palp two-segmented. Second segment spatulate. Two tooth-bristles on third masticatory lobe smooth.

Male L5 (Fig. 3C, D) asymmetrical. Right palp basally wide, grading to finger-like end, with two sub-apical setae. Left palp distally narrower than right, with two sub-apical setae.

Female L5 (Fig. 3B) with long *b*- and *d*- setae, and one long *a*-seta.

L6 (Fig. 3E) five-segmented. First segment with *d1*-seta extending slightly beyond this segment. Setae *e* and *f* extending to ca. tips of second and third endopodal segments, respectively. Seta *g* exceeding beyond terminal segment with ~ 50% of length. Terminal segment with *h1*-seta conspicuously longer than *h3*-seta.

L7 (Fig. 3F) five-segmented. First segment with *d1*-, *d2*- and *dp*- setae. Setae *e* and *f* absent. Seta *g* long. Terminal segment with short *h1*-seta and long *h2*- and *h3*- setae.

UR (Fig. 3H) with tiny seta *Sa.* Claw *Gp* slightly shorter than claw *Ga.* Seta *Sp* slightly exceeding end of ramus.

Hemipenis (Fig. 3G) sub-ovate in outline. Lobe *a* tongue-like, with sub-quadrate distal end. M-process with triangular distal part. Lobe *b* shorter than lobes *a* and *h*, with sub-quadrate distal part on inner edge and triangular distal part on outer edge. Lobe *h* slightly shorter than lobe *a*, with rounded distal part. Bursa copulatrix elongated with long finger distally.

###### Remarks.

The genus *Pseudocandona* Kaufmann, 1900, with 72 species described to date, is the third most diverse genus of the non-marine ostracods after *Candona* Baird, 1845 and *Strandesia* Stuhlmann, 1888 (see Meisch et al. 2019). [Karanovic (2005, 2012) proposed an alternative taxonomic scheme where Pseudocandona is treated as a subgenus under the genus Typhlocypris Vejdovský, 1882 and it only contains the six species of the *compressa* group, but we follow Namiotko et al. (2014) who redefined the genus *Typhlocypris*.] The genus, as accepted at the moment, consists of five species groups (*caribbeana*, *compressa*, *prespica*, *rostrata*, and *zschokkei*) and some species with uncertain positions (Namiotko and Danielopol 2004; Meisch et al. 2019). This division is mostly based on the number of posterior setae on the second segment of Md palp. Species with 5+1+*β* setae, as well as with the *h1*-seta on the L7 being more than twice the length of terminal segment, are classified in the *compressa* group (Meisch 1996, 2000; Namiotko and Danielopol 2004), to which the present new species also belongs. Other species of this group are *P.albicans* (Brady, 1864), *P.compressa* (Koch, 1838), *P.insculpta* (G. W. Müller, 1900), *P.pratensis* (Hartwig, 1901), *P.regisnikolai* Karanovic & Petkovski, 1999, and *P.sucki* (Hartwig, 1901) (Karanovic and Petkovski 1999; Meisch 2000; Karanovic 2012). *Pseudocandonaalbicans* can be distinguished from the present species by a shorter seta that accompanies the group of the five setae on the Md palp (sub-equally long to the grouped setae in the present species), a much slenderer first endopodal segment of the L6, as well as a slenderer carapace in dorsal view (Meisch 2000). Male bristles on the A2 are absent in *P.insculpta* (Meisch 2000), which easily distinguishes it from the present species. No other species of the *compressa* group has the morphology of the male L5 and the Hp similar to *P.cheni*. In *P.compressa*, *P.insculpta*, and *P.pratensis*, the lob *h* is not distally inflated (albeit slightly curved in *P.pratensis*), their right L5 have slenderer trunks. The lobe *h* in *P.sucki* is very wide, while the lobe *b* is small (Meisch 2000). *Pseudocandonaregisnikolai* is much larger (females range between 1.33 and 1.4 mm and males are up to 1.53 mm) (Karanovic and Petkovski 1999). In addition, *P.regisnikolai* possesses only one dorsal seta on the basal segment of the A1, setae *t2* and *t3* on the male A2 are not transformed into bristles, the left prehensile palp of male is much slenderer than the right one, and the *Hp* bears a conspicuous lobe *g* (Karanovic and Petkovski 1999).

#### Subfamily Cyclocypridinae Kaufmann, 1900

##### Genus *Cyclocypris* Brady & Norman, 1889

###### ﻿ Cyclocypris pangi sp. nov.

Taxon classificationAnimaliaPodocopidaCandonidae

0EC6A51A-94AA-5CB9-8FA4-658FD72E8409

http://zoobank.org/98BB8DE9-79A3-4985-9EC3-EAE47971E91C

Figs 4 , 5 , 6

Cyclocypris sp. – Zhai et al. 2017: 485, fig. 8.

####### Type locality.

A pond (Y34, Table 1) in Beijing, China.

####### Type material.

***Holotype***: one male (dyzoc559). ***Allotype***: one female (dyzoc558). ***Paratypes***: two females (dyzoc560, dyzoc561) and two males (dyzoc562, dyzoc563). All from the type locality, dissected, valves preserved on the micropalaeontological slides.

####### Other material.

One female (dyzoc675), from the site Y30 (Table 1), with soft parts dissected and valves preserved on a micropalaeontological slide. Two undissected specimens (dyzoc816, dyzoc817), from the site Y30 (Table 1), preserved dry on the micropalaeontological slides.

####### Etymology.

This species is named in the honour of Prof. Qiqing Pang (Hebei GEO University, China) in recognition of his productive work on Mesozoic and Cenozoic ostracods since the 1960s.

####### Dimensions.

Male, *n* = 3, LV, length 520‒540 μm, height 355‒387 μm. Female, *n* = 4, LV, length 483‒558 μm, height 331‒390 μm.

####### Diagnosis.

**Figure 4.**
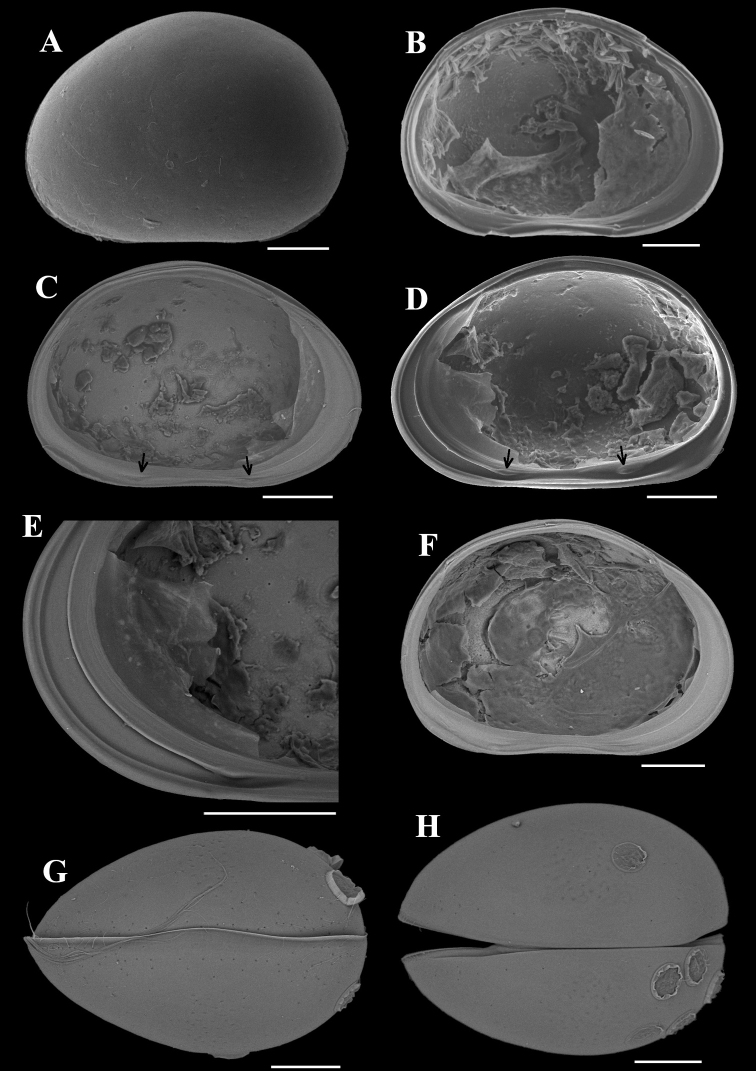
*Cyclocyprispangi* sp. nov. **A, B** female, dyzoc558 **A** outer view of LV**B** inner view of LV**C–E** female, dyzoc675 **C** inner view of LV, with sockets arrowed **D** inner view of RV, with pegs arrowed **E** anterior part of (D) showing details of calcified inner lamella **F** female, dyzoc560, inner view of LV**G** sex unknown, dyzoc816, ventral view of carapace **H** sex unknown, dyzoc817, dorsal view of slightly open carapace. Scale bars: 100 μm.

**Figure 5.**
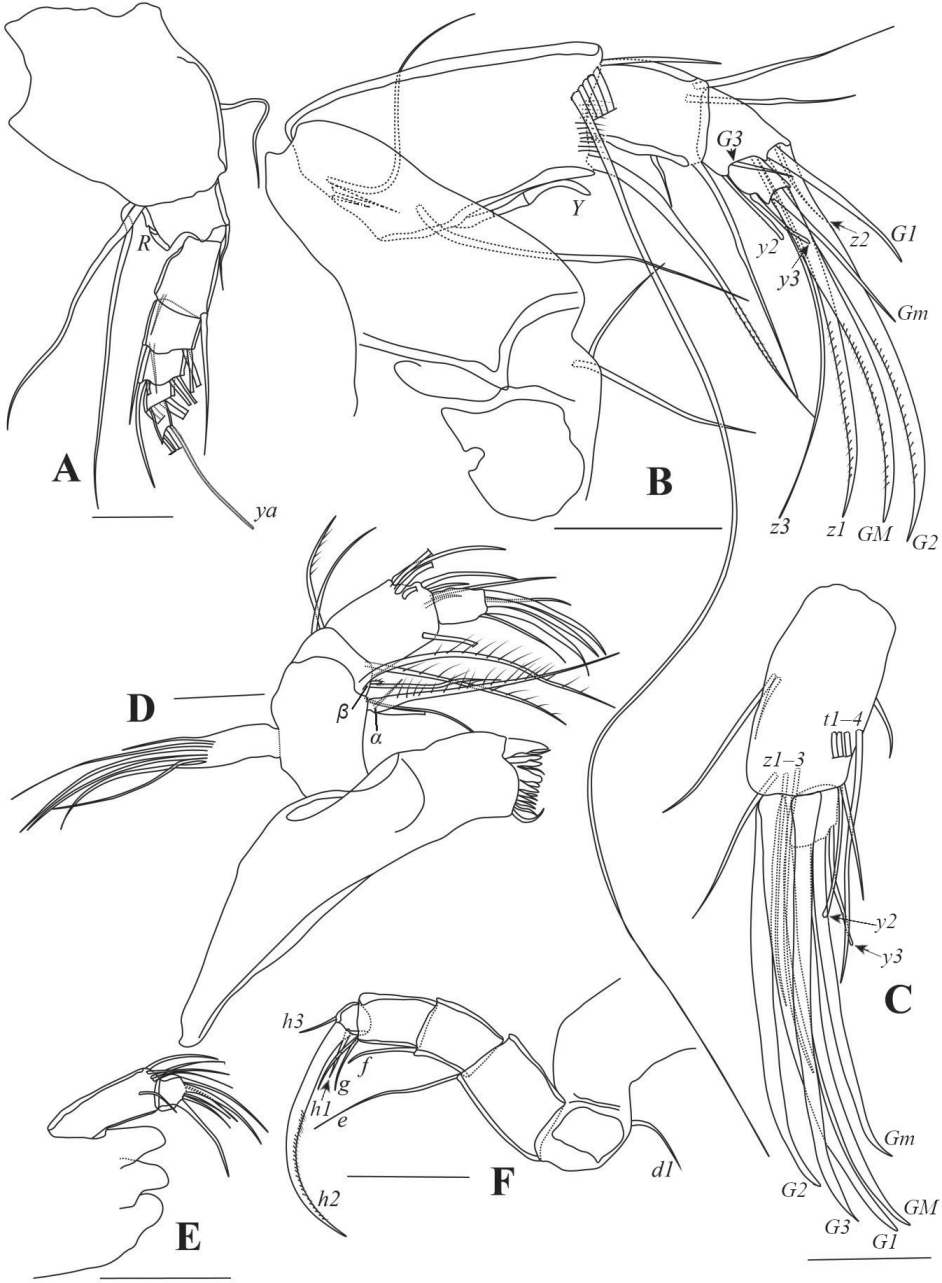
*Cyclocyprispangi* sp. nov. **A** male, dyzoc559 (holotype), A1**B** male, dyzoc559, A2**C** female, dyzoc558 (allotype), A2**D** female, dyzoc558, Md**E** female, dyzoc558, Mx**F** male, dyzoc559, L6. Scale bars: 100 μm.

**Figure 6.**
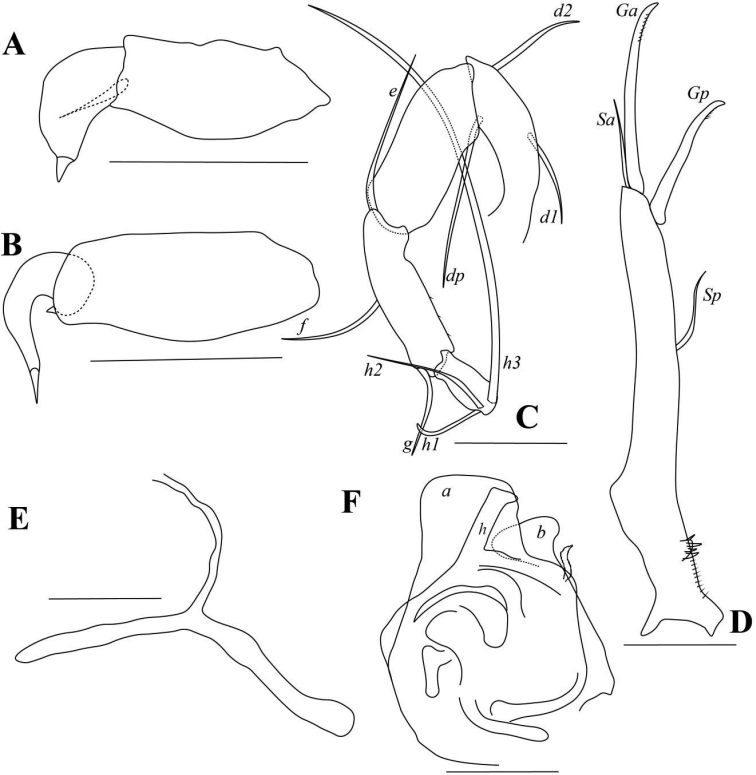
*Cyclocyprispangi* sp. nov. **A** male, dyzoc559, left L5**B** male, dyzoc559, right L5**C** female, dyzoc558, L7**D** female, dyzoc558, UR**E** female, dyzoc558, UR attachment **F** male, dyzoc559, Hp. Scale bars: 100 μm.

*Cyclocypris* species with intermediate-sized (Fig. 4A), dark-brown carapace. RV overlapping LV anteriorly and ventrally. A2 natatory setae exceeding terminal claws by 55% of length (Fig. 5B). Prehensile palps slightly asymmetrical, with sub-rectangular trunks, finger of left prehensile palp wider (Fig. 6A, B). L6*e*-seta exceeding end of terminal segment. L7 fourth segment with length almost twice of width, *h1* short and slightly curved, not “S-shaped” (Fig. 6C). Terminal claws of UR not reaching half-length of UR stem (Fig. 6D). Hp carrying S-shaped structure to interior side of lobe *b*, lobes *a* and *b* with wide distal end (Fig. 6F).

####### Description.

Carapace smooth. RV overlapping LV on all directions, and with one lobe-like expansion ventrally (Fig. 4G, H). Dorsal margin arched. Ventral margin almost straight in RV (Fig. 4D) and only slightly concave in LV (Fig. 4A‒C, F). Greatest height near middle length. Posterior end more rounded than anterior. Selvage peripheral along antero-ventral and postero-ventral margins (Fig. 4A). Two inner lists present on each valve (Fig. 4C‒F): interior one most pronounced on anterior margin of RV (Fig. 4E), weakly expressed on same position of LV (Fig. 4C, F); exterior one running close to selvage on both valves (Fig. 4C‒F) except postero-ventral part of LV (Fig. 4C, F). Antero- and postero-ventral parts of RV each with one blunt peg (arrows in Fig. 4D), corresponding to antero- and postero-ventral sockets on LV (arrows in Fig. 4C).

A1 (Fig. 5A) seven-segmented. First segment with one dorsal and two long ventral setae. Second segment with one dorso-apical seta and tiny Rome organ. Third segment with one medium-long dorso-apical seta and one short ventro-apical seta. Fourth segment with two long dorso-apical setae and two short ventro-apical setae. Fifth segment with two long dorso-apical setae, and one long and one short ventro-apical setae. Sixth segment with four long apical setae. Seventh segment with three long apical setae and aesthetasc *ya*.

Male A2 (Fig. 5B) five-segmented. Five long natatory setae extending beyond terminal claws with ~ 55% of their lengths. Sixth seta extending to end of next segment. Seta *z3* long, reaching to end of terminal claws. Seta *z1* well-developed and claw-like, slightly shorter than claws *G2* and *GM*. Claw *G1* short, not reaching mid-way of *G2*. Claw *G3* very small and seta-like. Claw *Gm* almost reaching mid-length of claw *GM*.

Female A2 (Fig. 5C) four-segmented. Seta *z1* shorter than setae *z2* and *z3*, exceeding mid-length of claw *G3*. Claws *G1*, *G3*, and *G2* progressively shorter. Claw *Gm* long, almost 80% length of claw *GM*.

Md (Fig. 5D) palp four-segmented. Seta *α* short and slim. Seta *β* very short and stout. Second segment with three long setae. Fourth segment with three claws and two setae.

Mx (Fig. 5E) palp two-segmented. First segment with four setae on outer apical edge and one seta in sub-apical position near outer edge. Second segment with three long and three short setae.

Male L5 (Fig. 6A, B) palps asymmetrical. First segment sub-rectangular. Left palp with bluntly rounded end and one sub-apical seta. Right palp slimmer than left, with one minute sub-apical seta. Distal end of right palp hook-like.

Female L5 (not shown, deformed in all specimens examined.) Exopod bearing five soft, thick rays. Other chaetotaxy structures difficult to discern.

L6 (Fig. 5F) five-segmented. First segment with *d1*-seta. Second segment with long *e*-seta, exceeding end of terminal segment. Third segment with *f*-seta slightly exceeding end of fourth segment. Fourth segment with two long *g*-setae, both exceeding beyond end of terminal segment. Fifth segment with short *h1*- and *h3*- setae and long claw *h2*.

L7 (Fig. 6C) four-segmented. First segment with *d1*-, *d2*- and *dp*- setae. Second segment with *e*-seta, not extending to end of third segment. Third segment with *f*-seta slightly exceeding end of this segment, and *g*-seta, slightly exceeding end of fourth segment. Fourth segment long, the length almost two times length of width with short *h1*- and *h2*- setae and long *h3*-seta.

UR (Fig. 6D) robust. Claw *Gp* exceeding mid-length of claw *Ga.* Seta *Sa* not reaching mid-length of claw *Ga.* Seta *Sp* short, not reaching base of claw *Gp.*UR attachment with two long branches.

Hemipenis (Fig. 6F) stout. Lobe *a* with wide, truncated distal end. Lobe *b* shorter than lobe *a* with wide distal end. Medial lobe *h* elongated, distally curved. Thin, slightly S-shaped structure with pointed end present to interior side of lobe *b*.

####### Remarks.

*Cyclocypris* is the third most diverse genus in Cyclocypridinae Kaufmann, 1900 and contains 21 species (Meisch et al. 2019). Except for *C.pusilla* Sars, 1895 reported from Afrotropical region (Sars 1895; Meisch et al. 2019), most *Cyclocypris* species are known from the Nearctic and Palaearctic regions. In China, three *Cyclocypris* species, *C.serena* (Koch, 1838), *C.globosa* (Sars, 1863), and *C.ovum* (Jurine, 1820), have been reported so far (Chen 1982; Huang 1986; Wang et al. 1995; Zhang et al. 2006; Kong et al. 2013). Majority of those species are known as fossils or sub-fossils, with the exception of *C.serena* (Kong et al. 2013).

The new species has some typical *Cyclocypris* characteristics. It is small, has a rounded carapace and long swimming setae on the A2; besides, it lacks male bristles on the A2 and has elongated terminal segment on the L7 and a long *g*-seta on the same appendage. The new species is somewhat similar to *C.serena* in terms of its size, the overlap of RV and LV, smooth surface of the valves, long swimming setae on the A2, rectangular basal segment of the L5, and short *h1*-seta on the L7. However, it can be distinguished from *C.serena* based on the following characters: (1) the lobes *a* and *b*, and the general shape of Hp, are much wider than in *C.serena* (Meisch 2000); (2) the length of the L6*e*-seta, the UR*Sa*- and *Sp*- setae, the number and length of the apical setae on both prehensile palps of the male L5, all differ from *C.serena*; (3) with respect to valve morphology, *C.serena* [0.58‒0.63 mm according to Meisch (2000)] is significantly larger than the new species, the inner list on the RV is less pronounced (Fuhrmann 2012), and the exterior inner list is absent. These differences in valve morphology would help distinguish the two species when dealing with sub-fossil / fossil material. The new species can be easily recognised, among the other 21 congeners, by the presence of a small, S-shaped process next to the lobe *b*, as well as a slender, distally curved lobe *h* (Fig. 6F).

### ﻿Family Cyprididae Baird, 1845

**Subfamily Eucypridinae Bronstein, 1947**

#### Genus *Tonnacypris* Diebel & Pietrzeniuk, 1975

##### ﻿ Tonnacypris rectangularis sp. nov.

Taxon classificationAnimaliaPodocopidaCyprididae

929CBCA1-73FE-5866-B83C-F15A90DEC506

http://zoobank.org/46D8213C-E160-4FDA-8CC6-D47BA32686E7

Figs 7 , 8 , 9

Tonnacypris sp. – Zhai et al. 2017: 488, fig. 11.

###### Type locality.

A small pond (Y11, Table 1) in Inner Mongolia, China.

###### Type material.

***Holotype***: one female (dyzoc575) dissected, valves preserved on the micropalaeontological slides. ***Paratype***: five females (dyzoc576‒580) dissected, valves preserved on the micropalaeontological slides. All from the type locality.

###### Other material.

Two undissected females (dyzoc819, dyzoc821) from the type locality, preserved dry on the micropalaeontological slides.

###### Etymology.

From the English word *rectangle*, referring to sub-rectangular valve shape in lateral view.

###### Dimensions.

Female, LV, *n* = 6, length 1800‒2030 μm, height 940‒1040 μm; RV, *n* = 4, length 1804‒2010 μm; height 980‒1060 μm.

###### Diagnosis.

**Figure 7.**
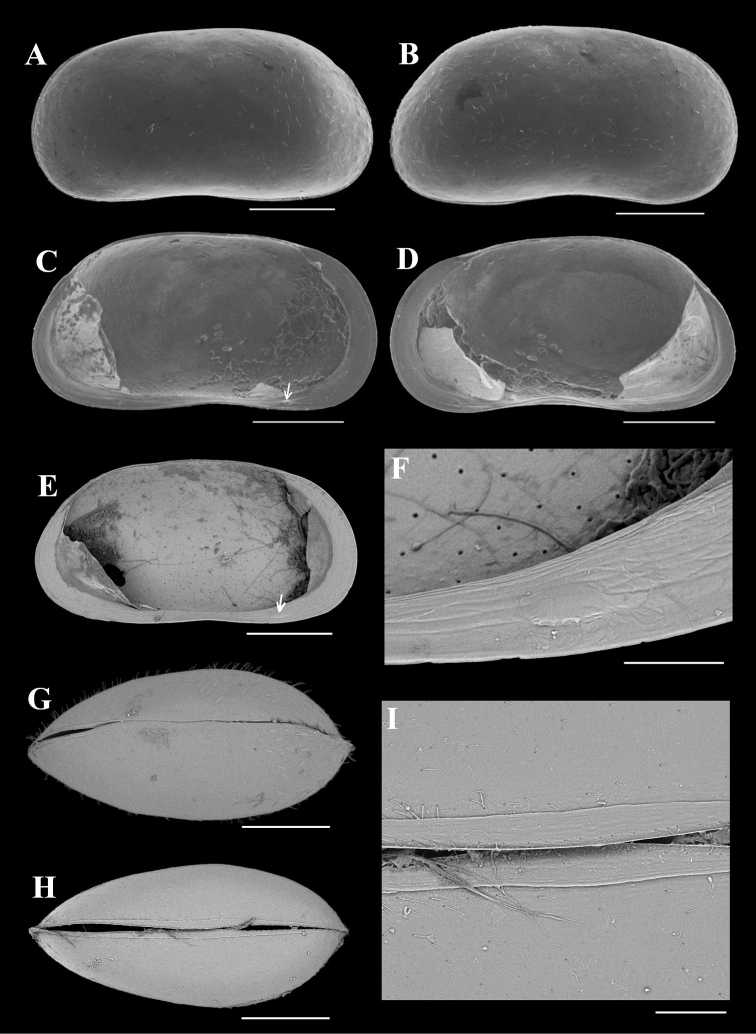
*Tonnacyprisrectangularis* sp. nov. Female **A–D** dyzoc575 (holotype) **A** outer view of LV**B** outer view of RV**C** inner view of LV, with peg arrowed **D** inner view of RV**E, F** dyzoc577 **E** inner view of LV, with peg arrowed **F** detail of peg in (**E**) **G** dyzoc821, slightly oblique-dorsal view of carapace **H, I** dyzoc819 **H** ventral view of carapace **I** detail of central part of (**H**), showing outer lists on both valves. Scale bars: 100 μm (**F, I**); 500 μm (**A–E, G, H**).

**Figure 8.**
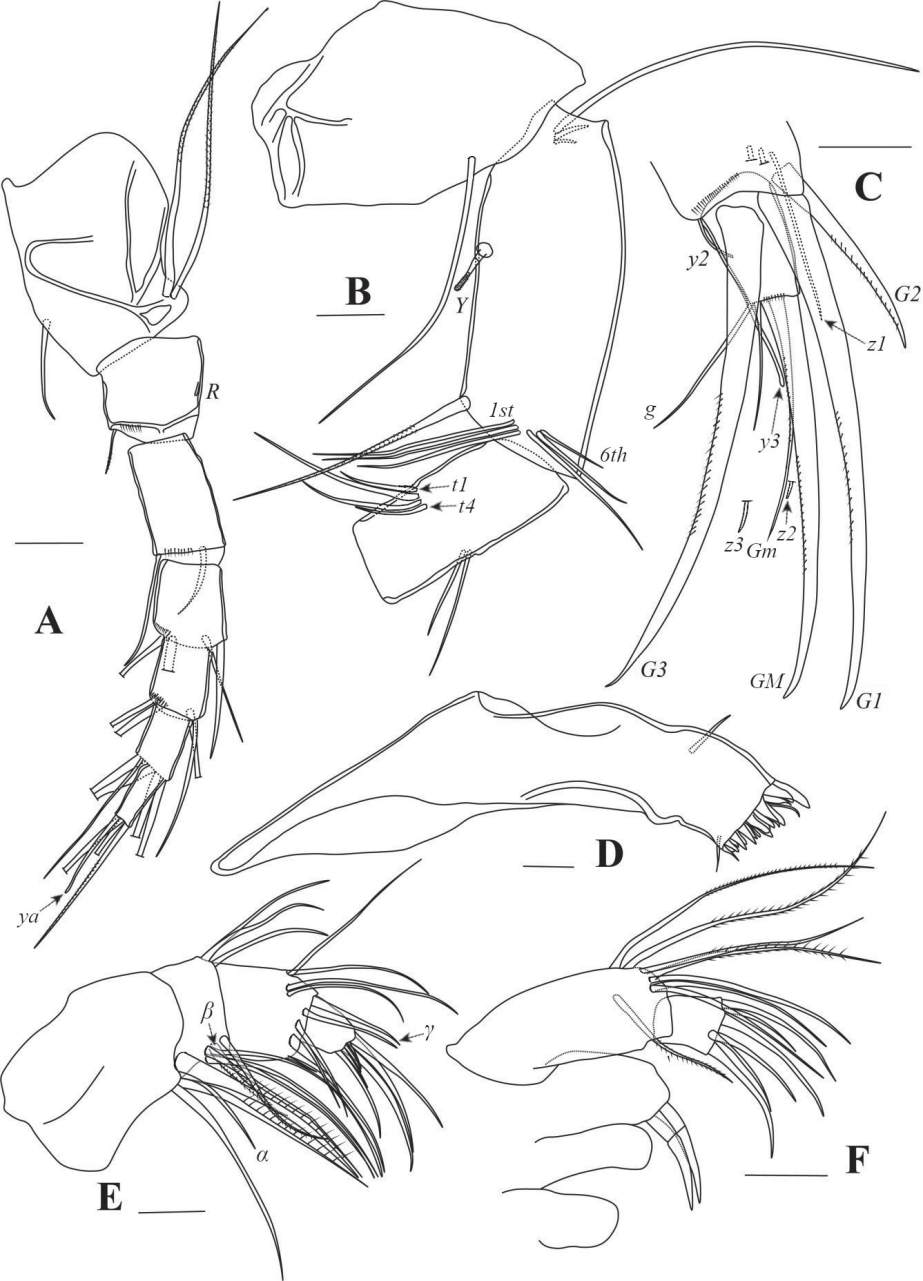
*Tonnacyprisrectangularis* sp. nov. Female. dyzoc575 (holotype) **A**A1**B** part of A2**C** part of A2**D**Md coxa **E**Md palp **F**Mx. Scale bars: 100 μm.

**Figure 9.**
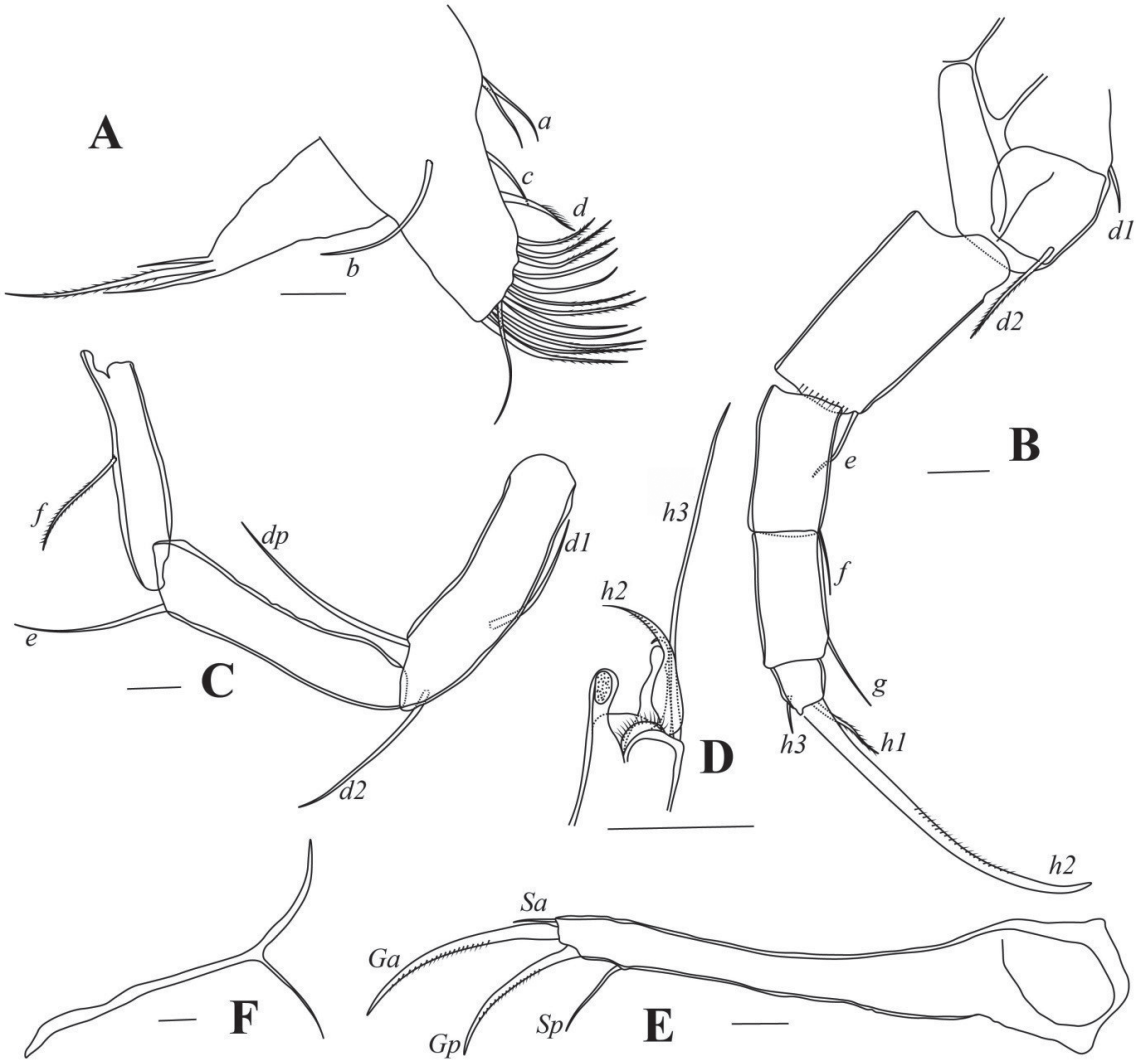
*Tonnacyprisrectangularis* sp. nov. Female **A** dyzoc580, L5**B** dyzoc580, L6**C** dyzoc580 part of L7**D** dyzoc580, part of L7**E** dyzoc575, UR**F** dyzoc575, UR attachment. Scale bars: 100 μm.

Carapace sub-rectangular, dorsal margin sub-parallel to ventral or slightly inclined anteriorly. Peg present on antero-ventral part of LV (Fig. 7C, E, F). Natatory setae on A2 reduced, first and second setae approximately in same lengths, others increasing in lengths towards anterior edge (Fig. 8B). Claw *G2* on A2 short, not reaching to middle length of claw *G3* (Fig. 8C). Second segment of Md palp with 3+1+*β* setae at interior side (Fig. 8E). Mx palp with terminal segment slightly spatulate. Two tooth-bristles on third masticatory lobe of Mx smooth (Fig. 8F). Length ratio between *d1*- and *d2*- setae on L6 0.41 (Fig. 9B).

###### Description.

Valves (Fig. 7) sub-rectangular, dorsal margin sub-parallel to ventral or slightly inclined anteriorly. When inclined, greatest height posterior of mid-length. Dorsal margin slightly arched posteriorly. Ventral margin concaved (Fig. 7A‒E). Anterior end more rounded than posterior. Calcified inner lamella wider anteriorly than posteriorly. Peg present on antero-ventral part of LV (Fig. 7C, E, F). Valve surface smooth, with setae. Carapace sub-ovate in dorsal / ventral view (Fig. 7G, H), with greatest width behind mid-length. Each valve with one outer list running through anterior and ventral margins (Fig. 7G, H, I).

A1 (Fig. 8A) seven-segmented. First segment with one short dorsal and two long ventral setae. Second segment with one short dorso-apical seta and a tiny Rome organ. Third segment with one comparative long dorso-apical seta and one short ventro-apical seta. Fourth segment with two long dorso-apical and two short ventro-apical setae. Fifth segment with two long dorso-apical setae and two setae (one long and one short) ventrally. Sixth segment with four long apical setae. Seventh segment with two long setae, one short seta and aesthetasc *ya*.

A2 (Fig. 8B, C) four-segmented. Natatory setae reduced, first and second setae almost in same lengths, others decreasing in lengths towards anterior edge. Claws *G1* and *G3* almost in same lengths. Claw *G2* short, not reaching mid-length of claw *G3*. Claw *Gm* slim, reaching mid-length of claw *GM*.

Md coxa (Fig. 8D) elongated and arched, with masticatory processes on interior end. Palp (Fig. 8E) four-segmented. Seta *α* long and slim. Seta *β* hirsute. Second segment with 3+1+*β* setae on interior side. Seta *γ* slim and smooth.

Mx (Fig. 8F) palp two-segmented. First segment with seven setae on outer apical edge and one seta in sub-apical position near outer edge. Second segment slightly spatulate with three long and three short setae. Two tooth-bristles on third masticatory lobe smooth.

L5 (Fig. 9A) with two *a*-setae, one long *b*-seta, one short *c*-seta and one hirsute *d*-seta.

L6 (Fig. 9B) five-segmented. Seta *d1* slightly shorter than half length of seta *d2*. Setae *e* and *f* short, not reaching end of next segment. Seta *g* long, slightly exceeding end of terminal segment. Seta *h1* longer than seta *h3*.

L7 (Fig. 9C, D) first segment with *d1*-, *d2*- and *dp*- setae. Second segment with *e*-seta not reaching end of third segment. Third segment medially with *f*-seta almost reaching end of this segment. Pincer organ typical of the genus, with comparatively long *h3*-seta and slender, gently curved *h2*-seta.

UR (Fig. 9E) with tiny seta *Sa.* Claw *Gp* exceeding half-length of claw *Ga.* Seta *Sp* slightly exceeding end of stem. UR attachment (Fig. 9F) with two long branches.

###### Remarks.

The genus *Tonnacypris* was first established with the fossil species *Tonnacyprisloessica* Diebel & Pietrzeniuk, 1975. *T.rectangularis* sp. nov. can be identified as belonging to this genus by the presence of peg on the LV [although not all specimens of this genus have pegs, see e.g., Peng et al. (2021)], presence of the *c*-seta on the L5, a short *d1*- and long *d2*- setae on the L6. There are nine extant species in this genus (Meisch et al. 2019). Among these, *T.angulata* Yang, 1985 has been described based on valves alone (Huang et al. 1985), and its generic assignment remains uncertain (Van der Meeren et al. 2009). The new species can be readily distinguished from all other extant congeners by the valve morphology (e.g., long and sub-parallel or anteriorly sloping dorsal margin, and narrow anterior calcified inner lamella) alone, but its soft parts offer additional diagnostic information. According to the length of the swimming setae on the A2, this species was the most similar to *T.mazepovae*Van der Meeren et al., 2009. But in *T.rectangularis* the surface of the carapace and the tooth-bristles on Mx are smooth, while in *T.mazepovae* the carapace surface is covered with superficial grooves and the tooth-bristles on the Mx are serrated (Van der Meeren et al. 2009). The present specimens are similar in valve shape and size to *T.tonnensis* (Diebel & Pietrzeniuk, 1975). However, in *T.tonnensis* the postero-ventral part of the valve is more narrowly rounded, and its A1 has a very long dorsal seta on the second segment (Van der Meeren et al. 2009: fig. 8). In addition, the UR attachment is not bifurcated in *T.tonnensis*. Among the fossil species, the type species *T.loessica* may resemble the new species in valve morphology (Fuhrmann 2012). Both species have sub-rectangular valves and their anterior calcified inner lamella is narrow. However, while the dorsal margin of *T.rectangularis* is sub-parallel to the ventral margin or is inclined anteriorly, the dorsal margin of *T.loessica* is sloping posteriorly. And the antero-dorsal part of both valves of *T.loessica* is angular, forming an antero-dorsal corner (cf. Fuhrmann 2012).

## ﻿Discussion

The world’s extant non-marine ostracods are distributed across all eight zoogeographical regions (Martens et al. 2008). According to the latest checklist (Meisch et al. 2019), 799 extant non-marine ostracods have been reported from the Palaearctic, the greatest number in all the regions at present. Inner Mongolia and Beijing are located in the central-eastern part of Palaearctic region. The three new species described in this study thus enrich the ostracod records for the PA region, as well as for these two provinces, where only 21 named species were reported until this study (Table 2). The number of ostracods known from the Beijing and Inner Mongolia (Table 2) is very low and a large area has remained unexplored. Further studies are needed to expand our knowledge due to the complex geography and the diversity of aquatic ecosystems in this region. This is especially true for Inner Mongolia, the third largest Chinese province, which occupies the widest longitude range across the country (97°24'‒126°04'E) but has a very small human population, which could provide suitable conditions for the survival of endemic ostracods.

**Table 2.** Named species of extant ostracods recorded in Inner Mongolia and Beijing, northern China.

No.	Species name	Beijing	Inner Mongolia	Reference	Zoogeographical region
1	*Bradleycyprisvittata* (Sars, 1903)	✓		Zhai and Zhao 2014; Zhai et al. 2017	AU, OL, PA
2	*Candonaquasiakaina* Karanovic & Lee, 2012	✓	✓	Zhai and Zhao 2014; Zhai et al. 2017	PA
3	*Cyclocyprispangi* sp. nov.	✓	✓	this study	PA
4	*Cypridopsisvidua* (O.F. Müler, 1776)	✓		Zhai and Zhao 2014; Zhai et al. 2017	AT, AU, NA, NT, OL, PA, PAC
5	*Cyprisgranulata* Daday, 1898	✓		Yu 2014 [as *Cyprissubglobosa* Sowerby, 1840]	AT, AU, NA, OL, PA
6	*Eucyprispigra* (Fischer, 1851)	✓		Zhai and Zhao 2014; Zhai et al. 2017	PA
7	*Fabaeformiscandonaalexandri* (Sywula, 1981)		✓	Zhai and Zhao 2014; Zhai et al. 2017	PA
8	*Fabaeformiscandonamyllaina* Smith & Kamiya, 2007	✓		Zhai and Zhao 2014; Zhai et al. 2017	PA
9	*Fabaeformiscandonasubacuta* (Yang, 1982)	✓		Zhai and Zhao 2014; Zhai et al. 2017	AU, NT, OL, PA
10	*Heterocyprisauricularis* Zhai & Zhao, 2014		✓	Zhai and Zhao 2014; Zhai et al. 2017	PA
11	*Heterocyprisvandouwei* (Brehm, 1923)	✓		Chen 1982	PA
12	*Heterocyprisincongruens* (Ramdohr, 1808)		✓	Zhai and Zhao 2014; Zhai et al. 2017	AT, AU, NA, NT, OL, PA, PAC
13	*Ilyocyprisangulata* Sars, 1903	✓		Zhai and Zhao 2014; Zhai et al. 2017	OL, PA
14	*Ilyocyprisinnermongolica* Zhai & Xiao, 2013		✓	Zhai and Xiao 2013; Zhai and Zhao 2014; Zhai et al. 2017	PA
15	*Ilyocyprismongolica* Martens, 1991		✓	Yu 2014; Zhai and Zhao 2014; Zhai et al. 2017	PA
16	*Ilyocyprissalebrosa* Stepanaitys, 1960	✓		Zhai and Zhao 2014; Zhai et al. 2017	NA, OL, PA
17	*Leucocytheremirabilis* Kaufmann, 1892		✓	Yu 2014	PA
18	*Limnocythereinopinata* (Baird, 1843)	✓	✓	Zhai and Zhao 2014; Zhai et al. 2017	AT, NA, PA
19	*Limnocytherestationis* Vávra, 1891	✓		Zhai and Zhao 2014; Zhai et al. 2017	AT, PA, OL
20	*Physocypriakraepelini* G.W. Müller, 1903	✓	✓	Zhai and Zhao 2014; Zhai et al. 2017	NA, OL, PA
21	*Plesiocypridopsisnewtoni* (Brady & Robertson, 1870)		✓	Zhai and Zhao 2014; Zhai et al. 2017	AT, PA, OL
22	*Pseudocandonacheni* sp. nov.		✓	this study	PA
23	*Potamocyprisvariegata* (Brady & Norman, 1889)	✓		Zhai and Zhao 2014; Zhai et al. 2017	NA, PA
24	*Tonnacyprisrectangularis* sp. nov.		✓	this study	PA
Total number of species		15	13	/	/

**Key**: AT, Afrotropical region; AU, Australasian region; NA, Nearctic region; NT, Neotropical region; OL, Oriental region; PA, Palaearctic region; PAC, Pacific Oceanic Islands. **Note**: We include here only the records with descriptions and/or illustrations of soft parts. Reports of sub-fossil valves (e.g., Zhai et al. 2013) are not included.

According to Martens and Segers (2009), ~ 90% of the 1936 non-marine ostracod species recognised at that time were endemic to one zoogeographical region, while only a few dozen species could be considered cosmopolitan. Yu et al. (2009) suggested that the number of Chinese endemic species should represent ~ 40% of the entire ostracods (including subfossil species) of this country. However, with the increasing knowledge on the extant non-marine ostracods of China (Kong et al. 2014; Zhai and Zhao 2014; Ma and Yu 2018, 2020; Peng et al. 2021), the ratio of endemic species seems to be declining. Among all the ostracods listed in Table 2, 12 species are endemic to the PA region: *Candonaquasiakaina* Karanovic & Lee, 2012, *Eucyprispigra* (Fischer, 1851), *Fabaeformiscandonaalexandri* (Sywula, 1981), *F.myllaina* Smith & Kamiya, 2007, *Heterocyprisauricularis* Zhai & Zhao, 2014, *H.vandouwei* (Brehm, 1923), *Ilyocyprisinnermongolica* Zhai & Xiao, 2013, *I.mongolica* Martens, 1991, *Leucocytheremirabilis* Kaufmann, 1892, in addition to the three new species described in this study. Another 12 ostracods are shared by the PA and other regions, among which *Cypridopsisvidua* (O.F. Müler, 1776), *Cyprisgranulata* Daday, 1898 [as *C.subglobosa* Sowerby, 1840 in Yu (2014)], and *Heterocyprisincongruens* (Ramdohr, 1808) are well known cosmopolitan species tolerant to a wide range of environmental conditions (Yu 2014).

The three new species also add to our knowledge on the geographical distribution of extant ostracods in general. *Cyclocyprispangi* is the first named ostracod species of the genus *Cyclocypris* reported from Beijing (Table 2). Similarly, *Pseudocandonacheni* and *T.rectangularis* are the first representatives of their respective genera recorded from Inner Mongolia, although Zhai et al. (2013) reported the valves of *Pseudocandona* sp. from Lake Hulun from the northern Inner Mongolia. The genus *Tonnacypris* has been rarely reported from China. The only named species of the genus reported from this country, *T.estonica* (Järvekülg, 1960), has been found from the Qinghai‒Tibet Plateau (Li et al. 2021; Peng et al. 2021). Mischke et al. (2003) found a fragment of the anterior part of RV of *Tonnacypris* (?) sp. from the Qilian Mountains from the northeastern margin of this plateau, the generic assignment of which is uncertain. Although members of this genus have been found in a number of sites in the PA region (e.g., Järvekülg 1960; Van der Meeren et al. 2009; Peng et al. 2021), their easternmost record was at 100°31'E in the northern part of Mongolia, represented by *T.mazepovae* (Van der Meeren et al. 2009). Thus, our study expands the known longitudinal range of the genus *Tonnacypris* eastwards, to ca. 116°45'E (Table 1).

Our detailed descriptions of the valves and carapaces of *P.cheni* (Fig. 1) and *C.pangi* (Fig. 4) provide clues for their identification from the sub-fossil and fossil assemblages. As stated above, the valves of *T.rectangularis* (Fig. 7) can be readily distinguished from its congeners by long, sub-parallel or anteriorly sloping dorsal margin and narrow anterior calcified inner lamella (see remarks of this species in the Taxonomy section). Previous studies (e.g., Mischke et al. 2010) suggested that the members of *compressa* group are difficult to identify to species level by using valve material only. The valves/carapace of *P.cheni* (Fig. 1), however, can be distinguished from other species in the group by the following carapace differences: *P.regisnikolai* is much longer (with females ranging between 1.33 and 1.4 mm and males being up to 1.53 mm) (Karanovic and Petkovski 1999); in dorsal view, *P.insculpta* is less inflated at the first 1/4 of its length, but more inflated at greatest width (slightly behind mid-length), and it is not compressed at the anterior end (Meisch 2000; Fuhrmann 2012); *P.compressa* is more laterally compressed in the dorsal view, and is less inflated at the first 1/4 (Meisch 2000; Fuhrmann 2012); *P.pratensis* is stouter, and the dorsal valve margin is conspicuously more inclined (Meisch 2000); *P.sucki* is more elongated in lateral view, with H/L ratio between 0.53 and 0.56 (*n* = 7, measured from Tafel, Germany (i.e., plate 42 in Fuhrmann 2012)), and in the dorsal view. both *P.pratenis* and *P.sucki* (see Meisch 2000 and Fuhrmann 2012), have a beak-shaped anterior end. The valves/carapaces of *C.pangi* (Fig. 4) resemble those of *C.serena* in lateral and dorsal/ventral outlines, but can still be distinguished from the latter. *Cyclocyprisserena* [0.58‒0.63 mm according to Meisch (2000)] is significantly larger than *C.pangi*, the inner list situated in the medial zone of the calcified inner lamella on the RV is less pronounced (Fuhrmann 2012), and the inner list running close to the valve margin is absent (see Fig. 4C‒F for the inner lists of *C.pangi*). These differences are helpful for distinguishing the two when dealing with the sub-fossil/fossil material. Considering intra-species morphological variations and possible loss of some fine-scaled structures such as the inner lists in the fossil material, however, some of the abovementioned differences may be obscure, and we suggest that geometric morphometric methods (e.g., Baltanás and Danielopol 2011; Namiotko et al. 2014) would be useful for the fine tuning of shell morphological characters.

## Supplementary Material

XML Treatment for Pseudocandona cheni

XML Treatment for Cyclocypris pangi

XML Treatment for Tonnacypris rectangularis
